# Dentes Sapientiæ

**Published:** 1875-02

**Authors:** 


*Dentes Sapientiæ.*—Dr. Jno. H. Coyle, of Thomasville, Ga. sends us two diminutive teeth of this class, which are of interest as anomalies, accompanied by the following history:

"In November last a young lady aged about 21 or 22, applied to me to have her upper teeth extracted with the view of having them replaced on a plate. I extracted the teeth and roots corresponding to 14, and found that neither of the Dentes Sapientiæ had been erupted. Eight days after the extraction. (all having been completed at one sitting,) she returned saying that I had left two roots. Upon examination I found the enclosed rudimentary Dentes Sapientiæ hanging to the gum, where the two second molars had been removed. I removed them with my fingers. You will observe one of them is perfect *in form* and apparently so in structure, while the other is almost destroyed by caries. If you deem them of sufficient interest you can place them in the Museum of the Baltimore College of Dental Surgery."

